# Supplementary material for: All-printed chip-less wearable neuromorphic system for multimodal physicochemical health monitoring
Source: Nat Commun. 2025 Jul 1;16:5689. doi: 10.1038/s41467-025-60854-7 (PMC12216833; doi:10.1038/s41467-025-60854-7)
Supplement: Supplementary file 1 — Supplementary Information [file 41467_2025_60854_MOESM1_ESM.pdf]

All-printed chip-less wearable neuromorphic system for multimodal physicochemical health monitoring

Yongsuk Choi[1,2,†], Peng Jin[1,†], Sanghyun Lee[1,3,†], Yu Song[1], Roland Yingjie Tay[1], Gwangmook Kim[1], Jounghyun Yoo[1], Hong Han[1], Jeonghee Yeom[1,2], Jeong Ho Cho[3,*], Dong-Hwan Kim[2,*] and Wei Gao[1,*]

[1]Andrew and Peggy Cherng Department of Medical Engineering, California Institute of Technology, Pasadena, CA 91125, USA.

[2]Department of Chemical Engineering, Sungkyunkwan University, Suwon 16419, Republic of Korea.

[3]Department of Chemical and Biomolecular Engineering, Yonsei University, Seoul 120-749, Republic of Korea.

[†]These authors contributed equally to this work.

*Email: weigao@caltech.edu, dhkim1@skku.edu, jhcho94@yonsei.ac.kr.

Table of Contents

Supplementary Fig. 1 | Schematic of fabrication procedure for the chip-less multimodal wearable neuromorphic system.

Supplementary Fig. 2 | Optical photos of the CSPINS.

Supplementary Fig. 3 | Bending flexibility evaluations of the wearable neuromorphic system.

Supplementary Fig. 4 | Fabrication of the synaptic biochemical sensors.

Supplementary Fig. 5 | Microscopic characterizations of plasma treatment on P3HT and SWCNTs surfaces.

Supplementary Fig. 6 | The crosslinking of ion-gel for synaptic transistors.

Supplementary Fig. 7 | Microscopic characterization of the inkjet-printed Au and PtNPs-modified Au.

Supplementary Fig. 8 | Electrical characteristics of synaptic transistors.

Supplementary Fig. 9 | Electrical characterizations for lactate synaptic sensors.

Supplementary Fig. 10 | Electrical characterizations for glucose synaptic sensors.

Supplementary Fig. 11 | Selectivity characterization of synaptic biochemical sensor.

Supplementary Fig. 12 | Stability study of synaptic lactate sensors.

Supplementary Fig. 13 | Influence of PBNPs on sensor performance.

Supplementary Fig. 14 | Synaptic biochemical sensors based on PtNPs and n-type In_2O_3 channel.

Supplementary Fig. 15 | Fabrication process of inkjet-printable MXene ink.

Supplementary Fig. 16 | Area-dependent synaptic characteristics of CBT calculating synapse.

Supplementary Fig. 17 | LTP/D characteristics of the synaptic device under varied temperatures.

Supplementary Fig. 18 | Voltage-dependent weight update characteristics for CBT-calculating synapse.

Supplementary Fig. 19 | Paired-pulse facilitation characteristics of the synapse.

Supplementary Fig. 20 | Preparation of the PDMS/MWCNT conductive sponge for pressure sensing.

Supplementary Fig. 21 | Calibration of conductive sponge-based pressure sensors.

Supplementary Fig. 22 | Heart rate monitoring using the conductive sponge sensor.

Supplementary Fig. 23 | Characterization of the inkjet-printed MWCNTs.

Supplementary Fig. 24 | Design and fabrication of amplifier circuits.

Supplementary Fig. 25 | Design and electrical characteristics of inkjet-printed voltage amplifier.

Supplementary Fig. 26 | Fabrication of inkjet-printed synaptic node circuit.
Supplementary Fig. 27 | Microscopic characterizations of the Nafion memristor.
Supplementary Fig. 28 | Electrical properties of the Nafion memristor with varying printing parameters.
Supplementary Fig. 29 | Characterization of Au/Nafion/Ag memristors and the corresponding synaptic node.
Supplementary Fig. 30 | Charging-discharging characteristics of the inkjet-printed node circuit.
Supplementary Fig. 31 | Bending flexibility evaluations of the wearable neuromorphic system.
Supplementary Fig. 32 | Electrical durability evaluations of the synapse.
Supplementary Fig. 33 | Configurations of input and output terminals for each synapse-node integrated device.
Supplementary Fig. 34 | Circuit diagram of wearable neuromorphic device for sepsis diagnosis.
Supplementary Fig. 35 | Cytotoxicity of the wearable neuromorphic device.
Supplementary Fig. 36 | Confusion matrix for a CSPINS validation.
Supplementary Fig. 37 | Estimated energy consumption of the synapse.
Supplementary Table 1 | List of material inks used in CSPINS fabrication.
Supplementary Table 2 | Reference skin and device temperatures measured with commercial thermometer.
Supplementary Table 3 | List of selected diagnosis case for device validation.
Supplementary Table 4 | Synaptic current and node outputs under various case inputs.
Supplementary Table 5 | List of selected confusion case for device validation.
Supplementary Table 6 | Synaptic current and node outputs under various case inputs.
Supplementary Table 7 | Evaluation of energy consumption of entire CSPINS.
Supplementary Table 8 | Comparison of power consumption of the CSPINS.
References

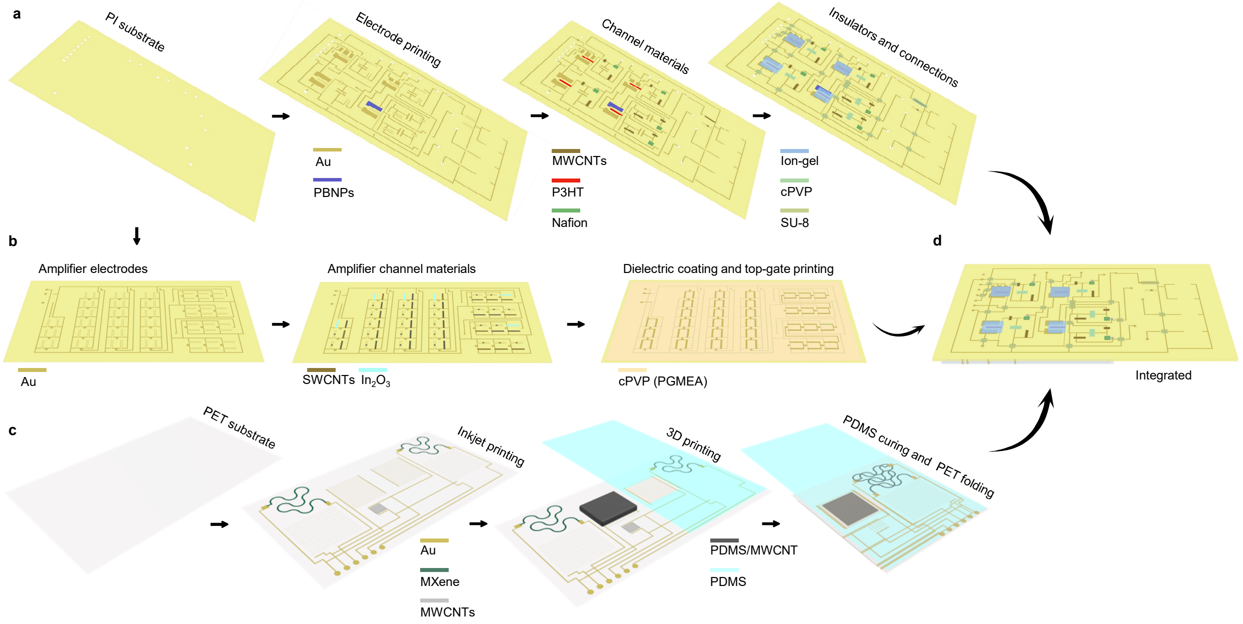

Supplementary Fig. 1 | Schematic of fabrication procedure for the chip-less multimodal wearable neuromorphic system. a,**b**, Fabrication of neuromorphic processing layer (**a**) and complementary amplifier circuits (**b**) on a polyimide (PI) substrate. PBNPs, Prussian blue nanoparticles; SWCNTs, semiconducting single-walled carbon nanotubes; MWCNTs, metallic multi-walled carbon nanotubes; P3HT, poly(3-hexylthiophene); cPVP, cross-linked poly(4-vinyl phenol). **c**, Fabrication process of multimodal physical sensors. PET, polyethylene terephthalate. **d**, Schematic of the assembled CSPINS. PDMS, polydimethylsiloxane. The layers are electrically connected through via holes drilled by a laser-cutting system.

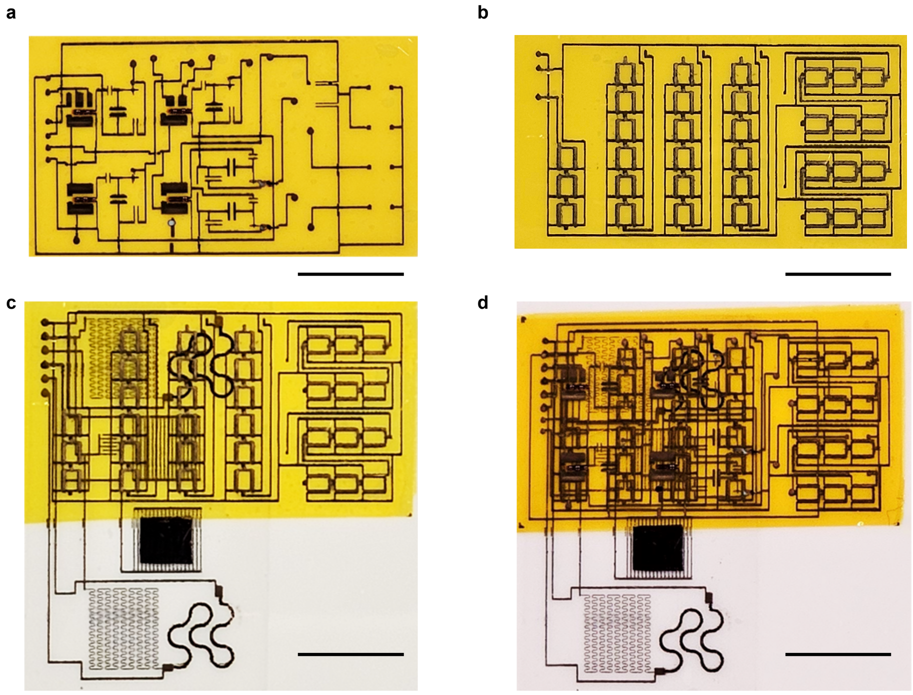

Supplementary Fig. 2 | Optical photos of the CSPINS. a, Optical images of the neuromorphic processing layer. **b**, Amplifier circuit layer. **c**, Assembled optical image of multimodal physical sensors with amplifier layers. **d**, Fully assembled CSPINS. Scale bars, 1 cm.

a b c

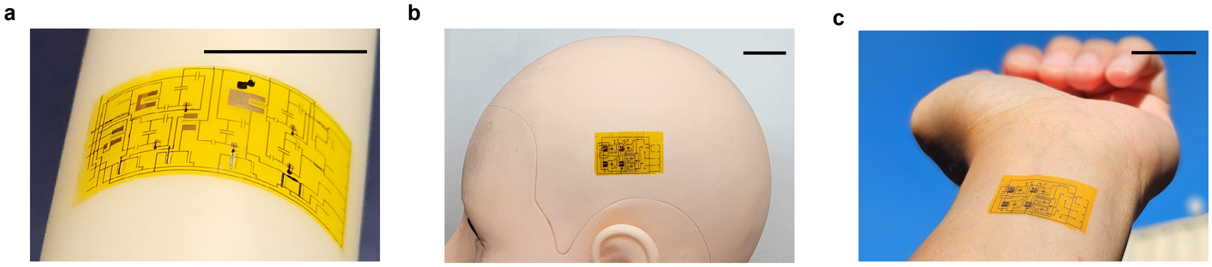

Supplementary Fig. 3 | Bending flexibility evaluations of the wearable neuromorphic system. **a-c**, Optical image of the wearable neuromorphic system conformally attached to the arbitrary surfaces such as round bar (**a**), mannequin's head (**b**) and wrist (**c**). Scale bars, 2 cm.

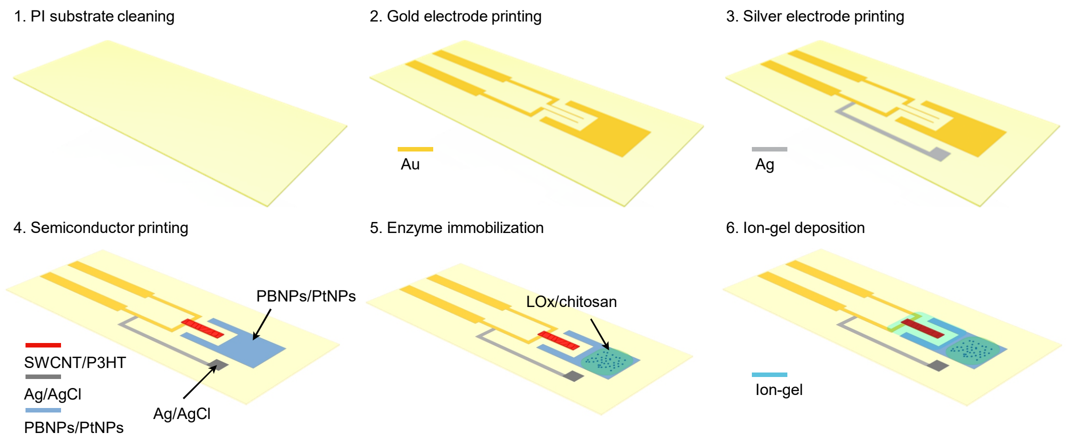

Supplementary Fig. 4 | Fabrication of the synaptic biochemical sensors. Au and Ag electrodes and interconnects are printed on a PI substrate. The Ag electrode is functionalized with $FeCl_3$ solution to form Ag/AgCl reference electrode, while the Au gate electrode is modified with PBNPs or Pt nanoparticles (PtNPs) through electroplating. The device is then finalized by sequentially depositing the synaptic channels, enzyme layer, and ion-gel.

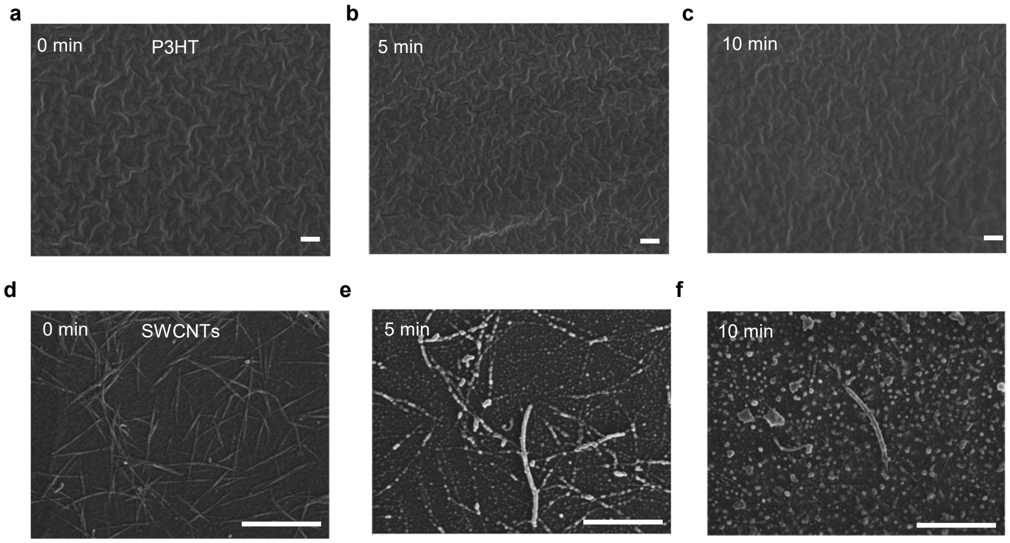

Supplementary Fig. 5 | Microscopic characterizations of plasma treatment on P3HT and SWCNT surfaces. a-c, Scanning electron microscopy (SEM) images of the top view of the printed P3HT surface prior and after O_2 plasma treatment with different exposure time between 0 to 10 min. **d-f,** SEM image of the printed SWCNTs surface prior and after O_2 plasma etching with different time from 0 to 10 min. Scale bars, 1 μm.

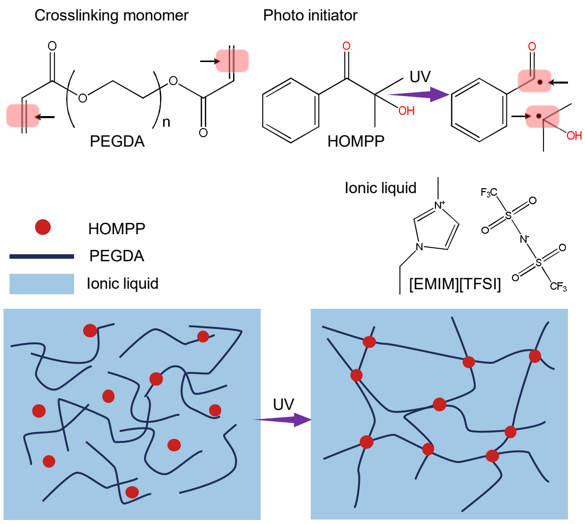

Supplementary Fig. 6 | The crosslinking of ion-gel for synaptic transistors. Crosslinking process of the ion-gel under UV exposure. PEGDA, polyethylene glycol diacrylate; [EMIM][TFSI], 1-ethyl-3-methylimidazolium bis(trifluoromethylsulfonyl)imide; HOMPP, 2-hydroxy-2-methylpropiophenone.

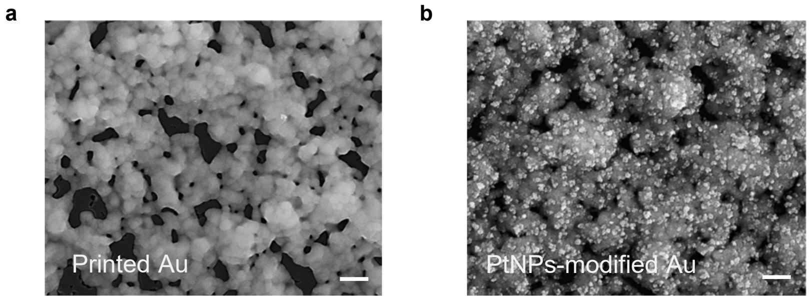

Supplementary Fig. 7 | Microscopic characterization of the inkjet-printed Au and PtNPs-modified Au. a, SEM images of the surface of printed Au. **b**, SEM image of the surface of PtNPs-modified Au. Scale bars, 400 nm.

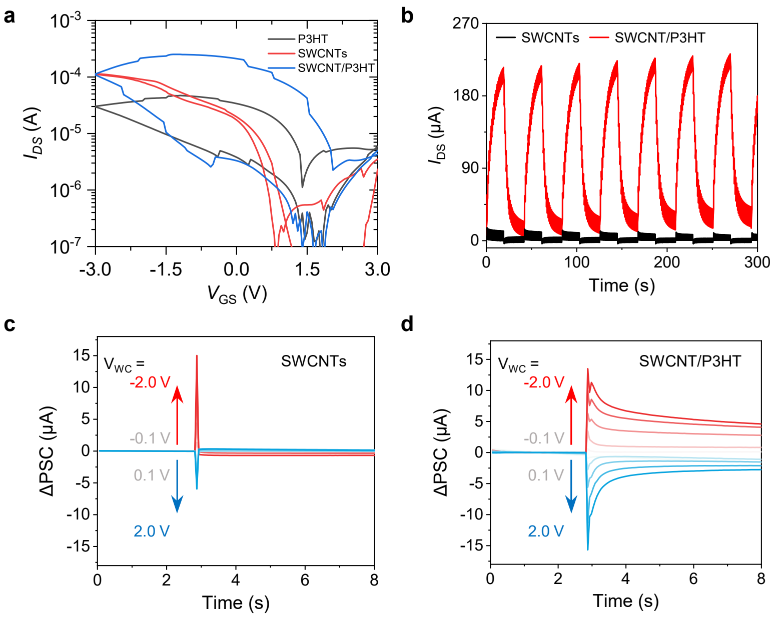

Supplementary Fig. 8 | Electrical characteristics of synaptic transistors. a, Transfer curves of transistors with different channel materials: P3HT, SWCNTs, and SWCNT/P3HT composite. In the SWCNT/P3HT device, SWCNTs serves as the primary conducting path for carriers, while P3HT provides memory functionality. **b–d,** Synaptic characteristics of transistors based on SWCNTs and SWCNT/P3HT semiconducting channels, including long-term potentiation and depression (LTP/D) of SWCNTs and SWCNT/P3HT transistors (**b**), excitatory/inhibitory post-synaptic currents (E/IPSC) of the SWCNTs transistor (**c**), and E/IPSC of the SWCNT/P3HT transistors (**d**).

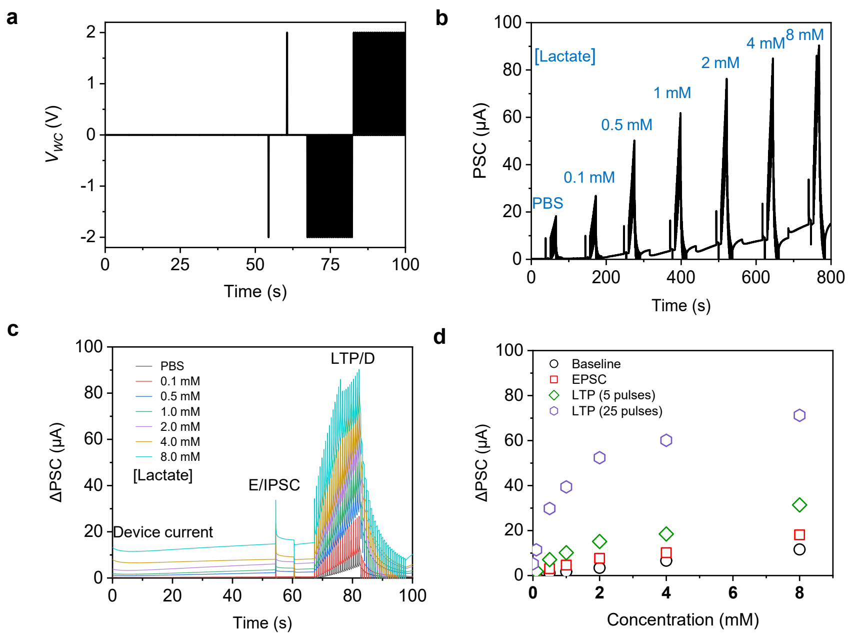

Supplementary Fig. 9 | Electrical characterizations for lactate synaptic sensors. a, Input voltage pulses applied to the gate electrode (t_{on}, 50 ms; period, 0.5 s) to generate synaptic currents. **b**, Real-time post-synaptic current (PSC) change of the synaptic device at various lactate concentrations (0–8 mM). **c**, Normalized PSC changes under varied lactate concentrations, displaying device base current, E/IPSC, and LTP/D. **d**, Extracted PSC values for base current, excitatory post-synaptic current (EPSC), and LTP at different weight updates as a function of lactate concentrations.

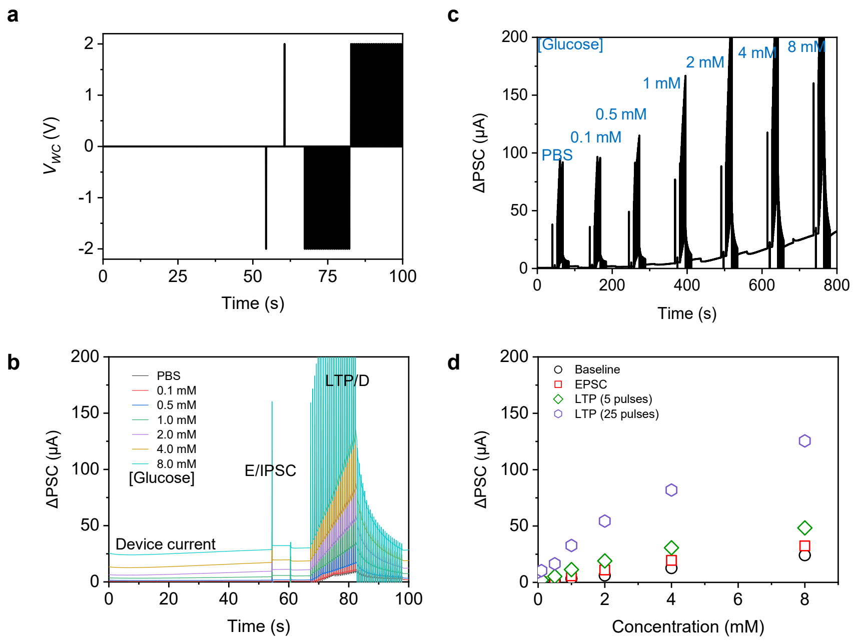

Supplementary Fig. 10 | Electrical characterizations for glucose synaptic sensors. a, Plot of input voltage pulses applied to the gate electrode (t_{on}, 50 ms; period, 0.5 s) to generate synaptic currents. **b**, Real-time PSC change of the synaptic device at various glucose concentrations (0–8 mM). **c**, Normalized PSC changes under varied glucose concentrations, displaying device base current, E/IPSC, and LTP/D. **d**, Extracted PSC values of base current, EPSC, and LTP at different weight updates as a function of glucose concentrations.

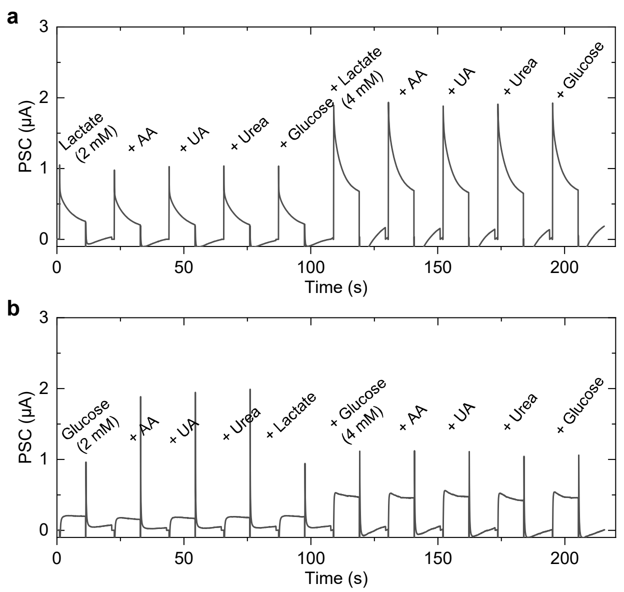

Supplementary Fig. 11 | Selectivity characterization of synaptic biochemical sensor. a, E/IPSC of the synaptic lactate sensor in the presence of various biochemical interferences: ascorbic acid (AA) (60 μM), uric acid (UA) (0.3 mM), urea (5 mM), and glucose (4 mM). **b**, E/IPSC of synaptic glucose sensor in the presence of various biochemical interferences: AA (60 μM), UA (0.3 mM), urea (5 mM), and lactate (4 mM).

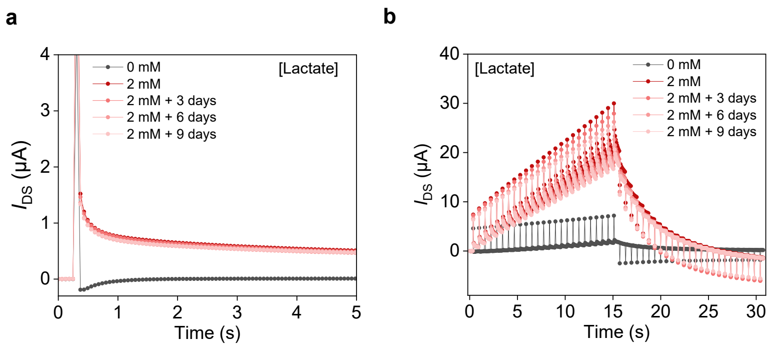

Supplementary Fig. 12 | Stability study of synaptic lactate sensors. a, EPSC of a synaptic lactate sensor in 0 and 2 mM lactate solutions. Tests in 2 mM lactate were repeated after 3, 6, and 9 days. **b**, LTP/D curves of a synaptic lactate sensor in 0 and 2 mM lactate solutions. Tests in 2 mM lactate were repeated after 3, 6, and 9 days.

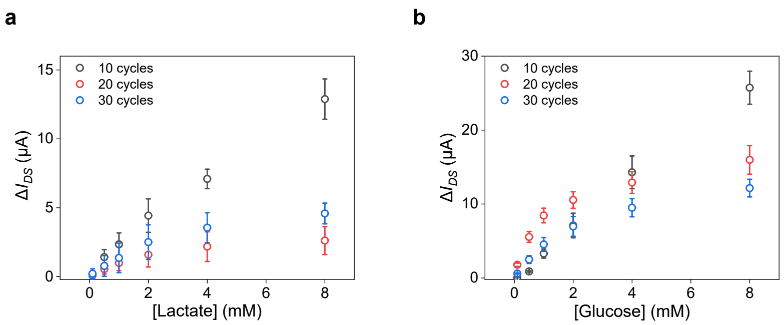

Supplementary Fig. 13 | Influence of PBNPs on sensor performance. a,**b,** Calibration curves of the synaptic lactate (**a**) and glucose (**b**) sensors with 10, 20, and 30 cycles of PBNPs electrodeposition.

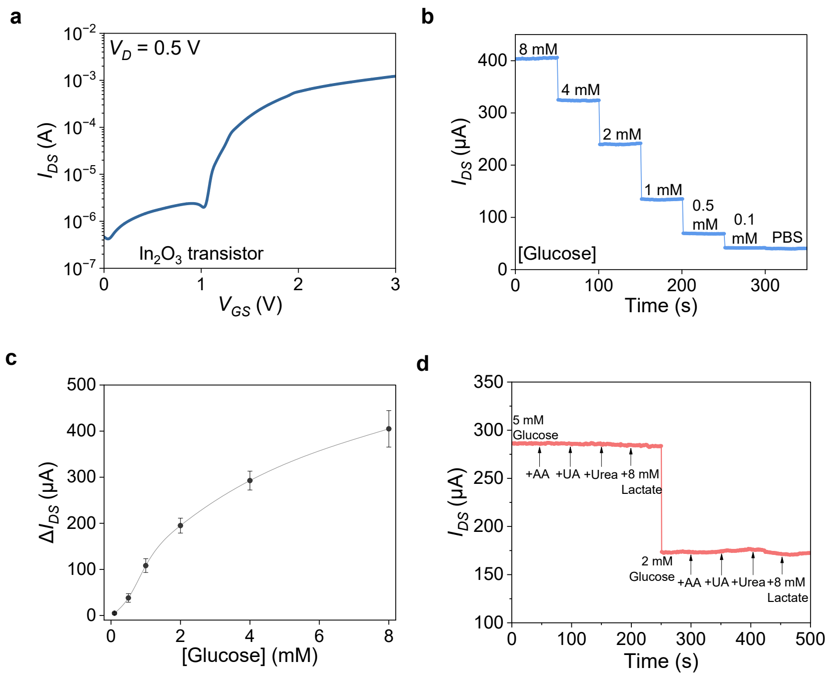

Supplementary Fig. 14 | Synaptic biochemical sensors based on PtNPs and n-type In_2O_3 channel. a, Transfer curve of the inkjet-printed In_2O_3 channel based transistor gated by ion-gel. The drain electrode is applied with 0.5 V. The gate electrode is applied with 1.2 V during glucose sensing measurement. **b**, Drain current of the In_2O_3 transistor-based glucose sensors under varying glucose concentrations. The In_2O_3 transistor gate electrode is modified with PtNPs and then with enzyme/chitosan. **c**, Response currents as a function of glucose concentration. The response currents are measured relative to the drain current when gate electrode is in the presence of PBS. **d**, Selectivity of the In_2O_3 transistor-based glucose sensors in the presence of interfering biochemicals including AA (60 μM), UA (300 μM), urea (5 mM), and lactate (8 mM).

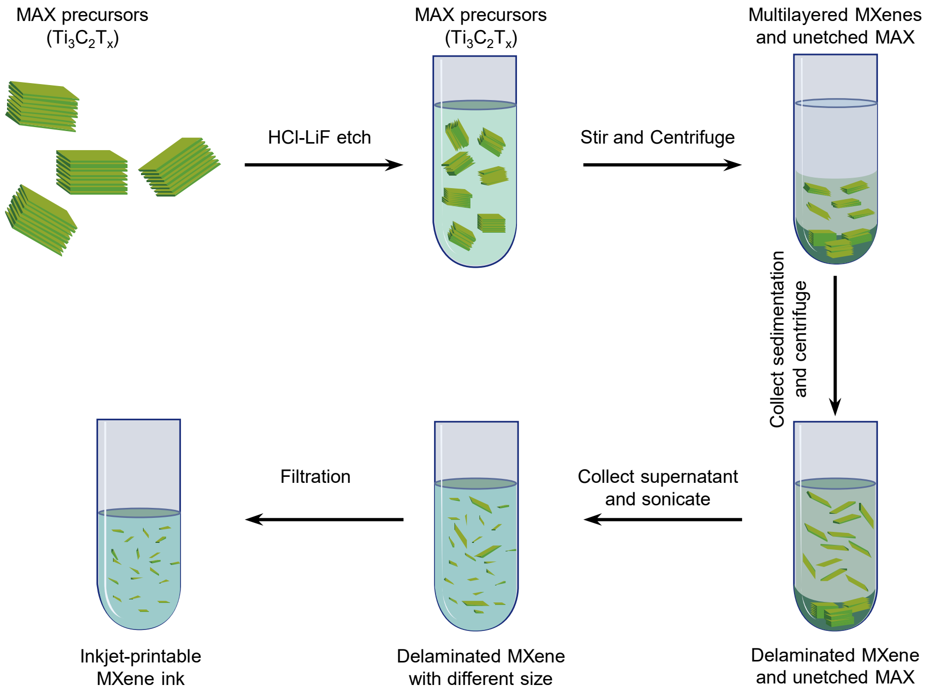

Supplementary Fig. 15 | Fabrication process of inkjet-printable MXene ink. a, Preparation of $Ti_3C_2T_x$ (MAX) precursor. **b**, Minimally intensive layer delamination etching of MAX precursor. **c**, Mixed colloid state containing multilayered MXene and unetched MAX. **d**, Shake and centrifuge process to collect delaminated MXene. **e**, Collection of the supernatant after centrifuge. **f**, Preparation of inkjet-printable MXene ink by mixing the MXene solution with ethylene glycol in a 4:6 ratio and tip-sonicating the mixture.

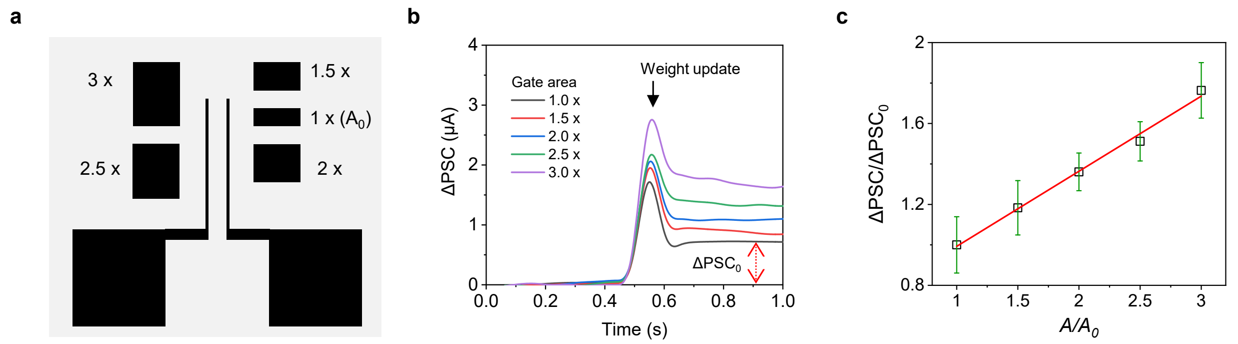

Supplementary Fig. 16 | Area-dependent synaptic characteristics of CBT calculating synapse. **a**, Schematic design of a multi-gated synaptic device to evaluate gate-area-dependent PSC. The relative gate electrode area was set to 1.5, 2, 2.5 and 3 times larger than the reference (1×, A_0). **b**, EPSC plots with a potentiation pulse (amplitude, -3 V; t_{on}, 50 ms; period, 0.5 s) applied at different gate electrodes. **c**, Extracted relative PSC values (PSC/PSC_0) as a function of the gate area ratio (A/A_0). PSC_0, EPSC operated by 1x gate electrode.

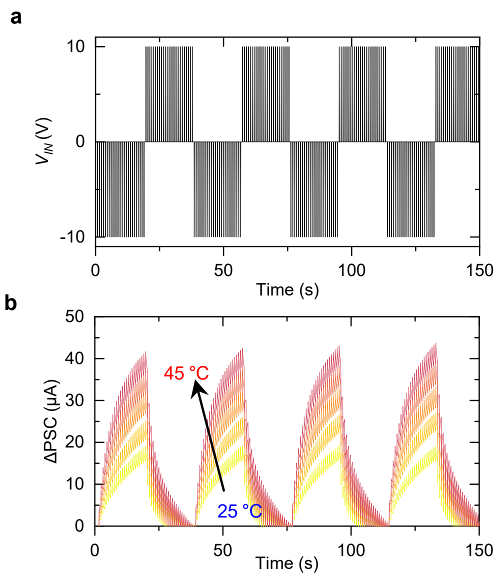

Supplementary Fig. 17 | LTP/D characteristics of the synaptic device under varied temperatures. a, Input voltage pulses applied to the temperature sensor part (t_{on}, 50 ms; period, 0.5 s) to generate synaptic currents. **b**, LTP/D curves of the synaptic device at different temperatures.

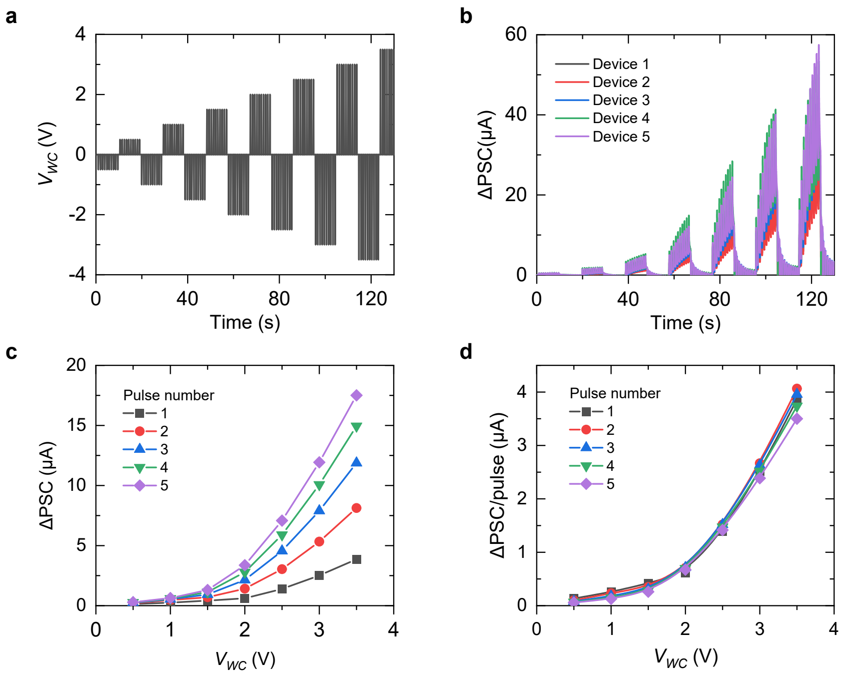

Supplementary Fig. 18 | Voltage-dependent weight update characteristics for CBT-calculating synapse. a, Plot of input voltage pulses applied to the gate electrode (t_{on}, 50 ms, period, 0.5 s) to generate synaptic currents, with weight update pulse amplitudes varied from ±0.5 to ±3.5 V. **b**, LTP/D characterization of synaptic under varying voltage amplitudes. **c**, The average PSC change from weight updates 1 to 5 as a function of voltage amplitude. **d**, Normalized PSC change as a function of the weight update number.

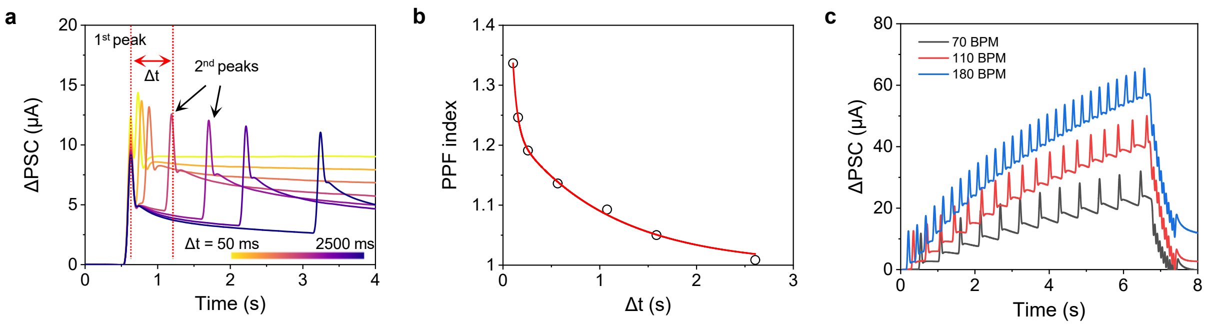

Supplementary Fig. 19 | Paired-pulse facilitation characteristics of the synapse. a, PSC plot of the synapse under two weight-update pulses (t_{on}, 50 ms; amplitude, -3 V) with different time lags (Δ*t*). **b**, Paired-pulse facilitation (PPF) index as a function of time lags. PPF index refers to the ratio between the first and second peaks of ΔPSC. Red curve represents exponential decaying fitting. **c**, LTP characteristics of synapse under different weight-update frequencies.

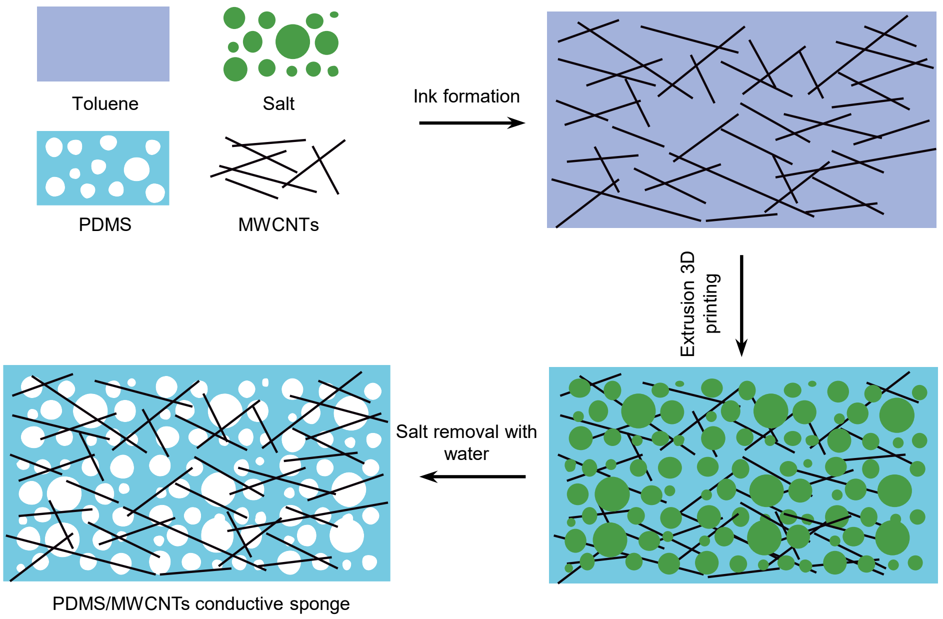

Supplementary Fig. 20 | Preparation of the PDMS/MWCNT conductive sponge for pressure sensing.

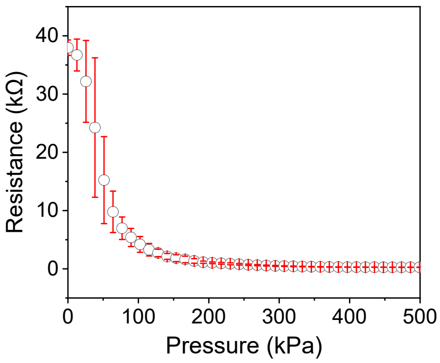

Supplementary Fig. 21 | Calibration of conductive sponge-based pressure sensors. Error bars represent s.d. of the mean from 5 different sensors.

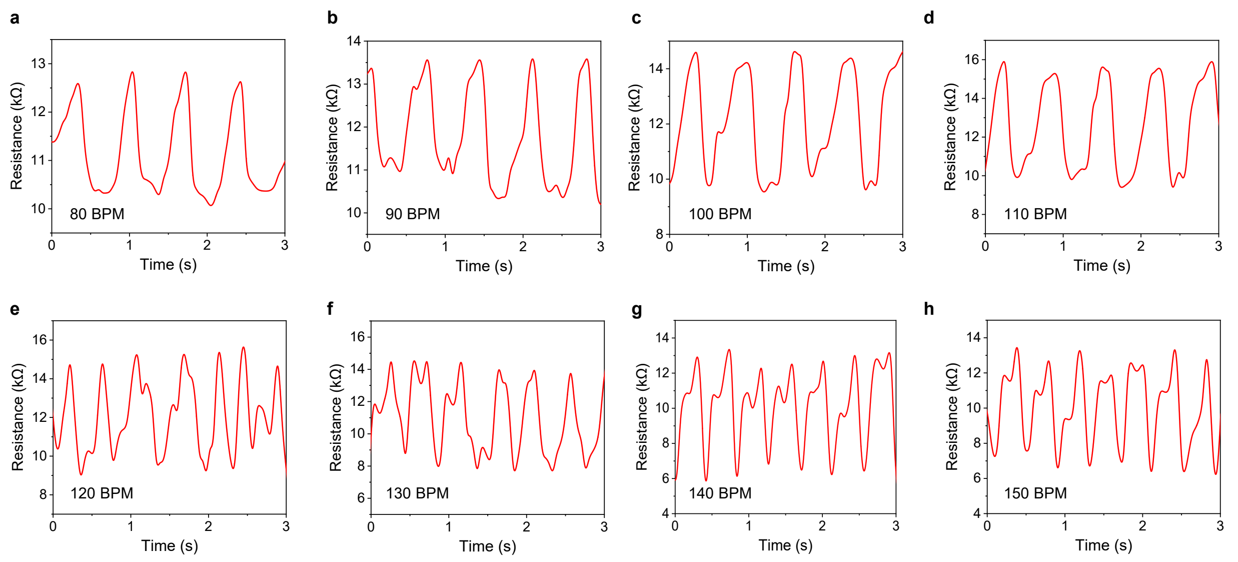

Supplementary Fig. 22 | Heart rate monitoring using the conductive sponge sensor. a–h, Sensor response under different heart rates. Labelled heart rate value was taken with a commercial heart rate monitor.

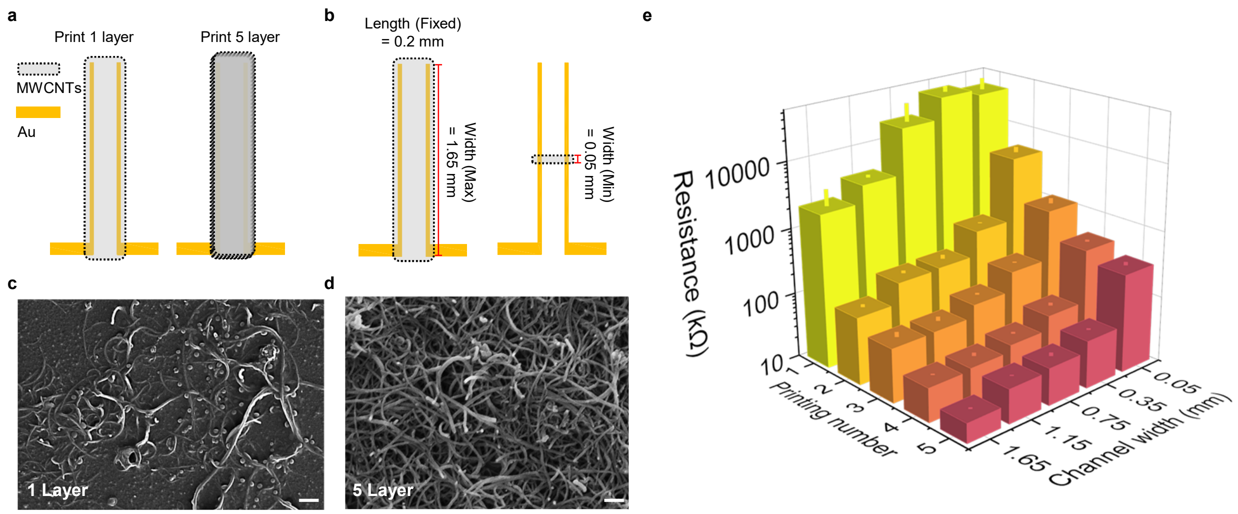

Supplementary Fig. 23 | Characterization of the inkjet-printed MWCNTs. a,**b**, Schematic designs of the MWCNTs resistors with varying printing cycle numbers (**a**) and channel widths (**b**). **c**,**d**, SEM images of the MWCNTs prepared via one (**c**) and five (**d**) printing cycles. **e**, Dependence of the resistance values of MWCNTs resistors on printing cycle numbers and channel widths.

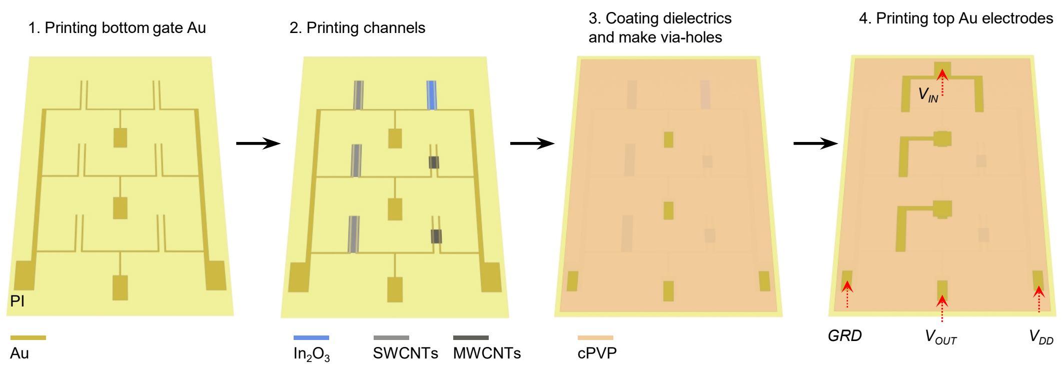

Supplementary Fig. 24 Design and fabrication of amplifier circuits.

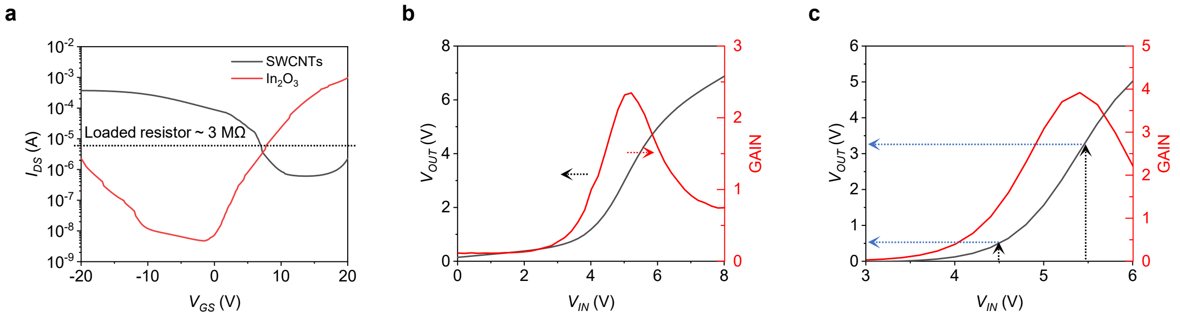

Supplementary Fig. 25 | Design and electrical characteristics of inkjet-printed voltage amplifier. a, Transfer characteristics and loaded resistor level of the SWCNTs, In_2O_3 transistors and MWCNTs resistors. V_D = 10 V, SWCNTs, single-walled carbon nanotubes; MWCNTs, multi-walled carbon nanotubes. **b,c,** Plots of input (V_{IN}), output (V_{OUT}), and corresponding gain of SWCNT/ In_2O_3 amplifier (**b**) and 3-stage amplifier circuits (**c**).

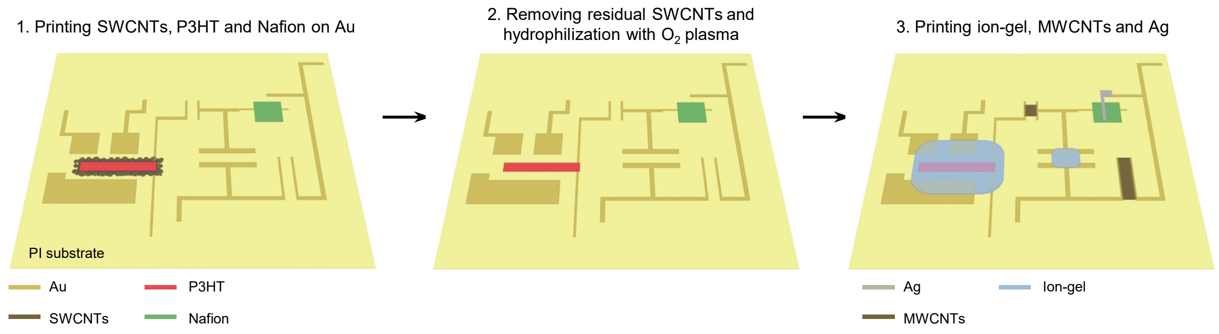

Supplementary Fig. 26 | Fabrication of inkjet-printed synaptic node.

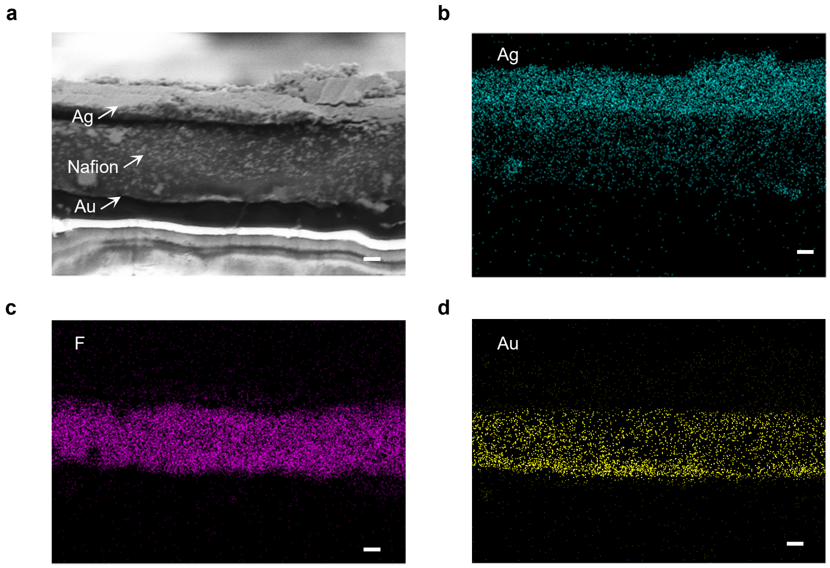

Supplementary Fig. 27 | Microscopic characterizations of the Nafion memristor. a–d, Cross-sectional SEM image of the Ag/Nafion/Au memristor device (**a**), and the corresponding energy dispersive X-ray spectroscopy (EDS) mapping of Ag (**b**), fluorine (F) (present in Nafion) (**c**), and Au (**d**). Scale bars, 1 μm.

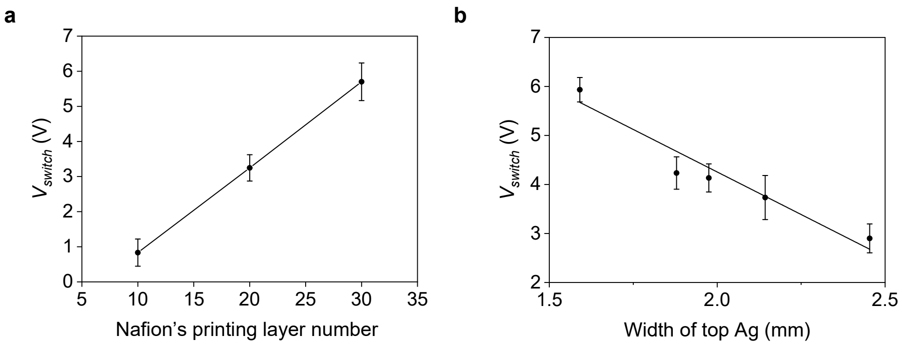

Supplementary Fig. 28 | Electrical properties of the Nafion memristor with varying printing parameters. a,**b**, Switching voltage of the memristors as a function of the Nafion’s printing layer number and the width of top Ag electrode (**b**).

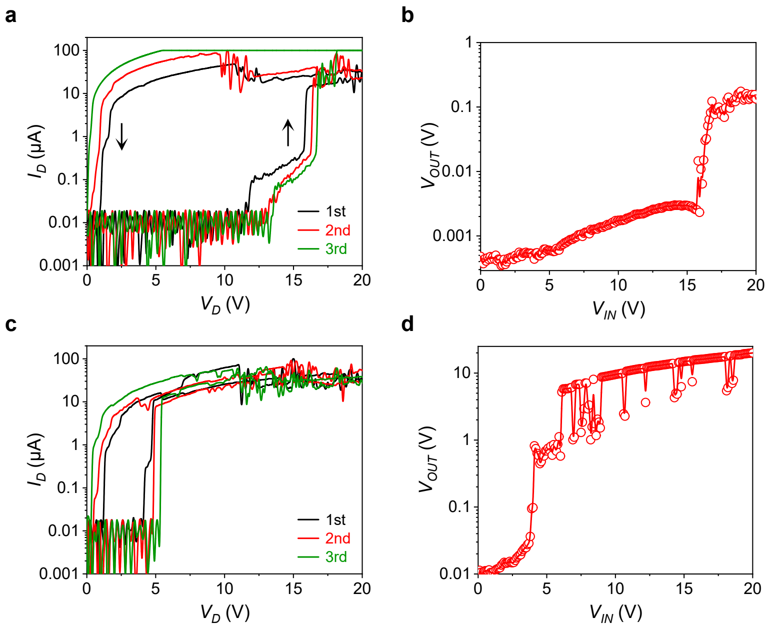

Supplementary Fig. 29 | Characterization of Au/Nafion/Ag memristors and the corresponding synaptic node. **a**, Current plots of Au/Nafion/Ag memristor under three repeated cycles of applied voltage. **b**, Input-output voltage plot of node circuit based on memristors with a switching voltage of ~16 V. **c**, Current plots of Nafion/Ag memristor with a switching voltage ~ 5 V. **d**, The input-output voltage plot of the corresponding synaptic node based on memristors with a switching voltage ~ 5 V.

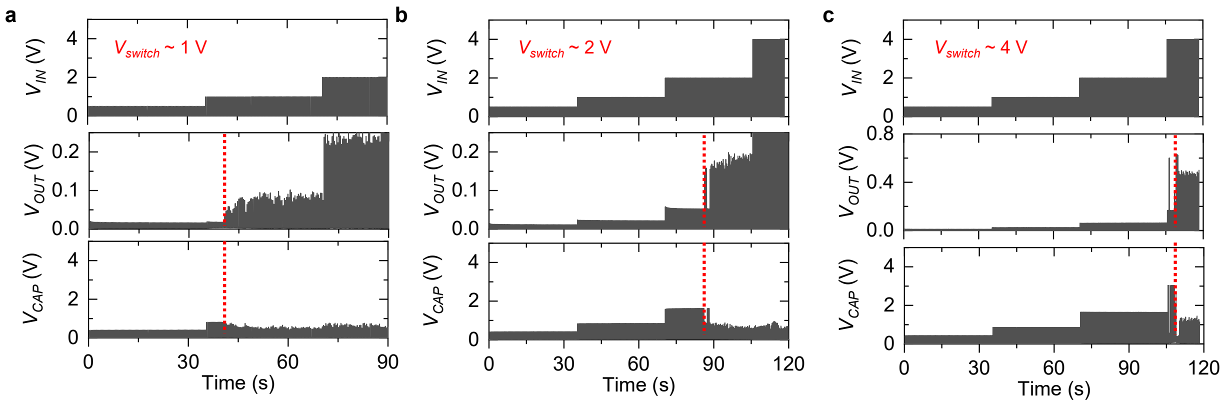

Supplementary Fig. 30 | Charging-discharging characteristics of the inkjet-printed node circuit. a–c, Plots of input (V_{IN}), output (V_{OUT}), and capacitor voltage (V_{CAP}) from the inkjet-printed node circuit with switching voltages of ~1 V (**a**), ~ 2 V (**b**) and ~4 V (**c**).

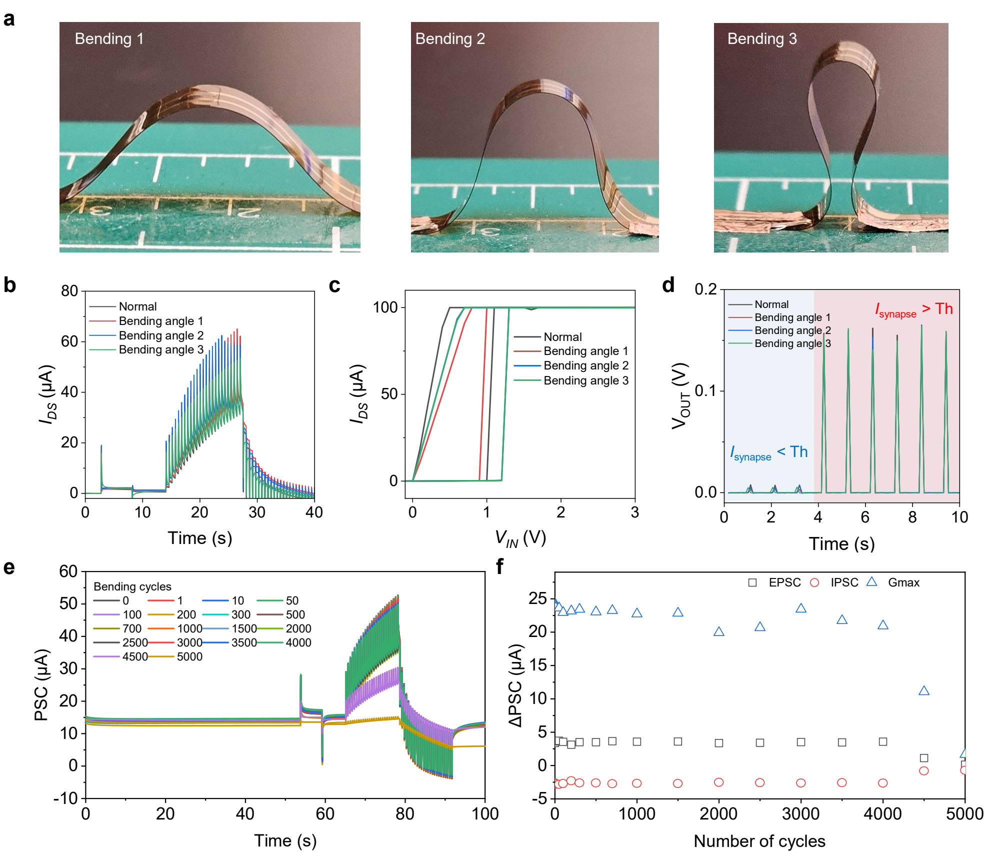

Supplementary Fig. 31 | Bending flexibility evaluations of the wearable neuromorphic system. **a**, Optical images of the wearable neuromorphic system under different bending angles. **b**, Characterization of synaptic currents of artificial synapse unit under different bending angles. **c**, Characterization of Nafion memristors under different bending angles. **d**, Characterization of synapse-node integrated circuit under different bending angles. Th, threshold. **e**, Real-time changes in PSC under repeated bending cycles. **f**, Evolution of EPSC, IPSC, and maximum conductance (G_{max}) during repeated bending tests.

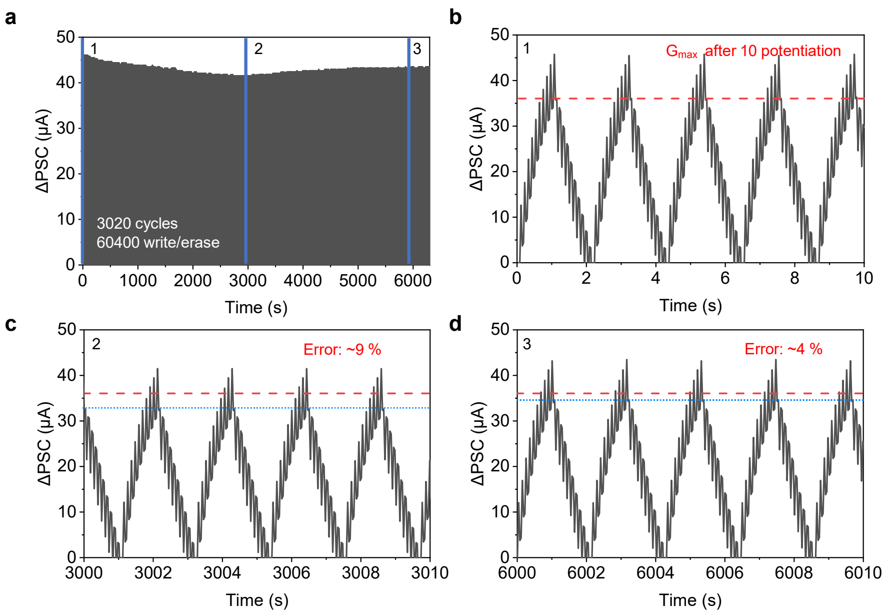

Supplementary Fig. 32 | Electrical durability evaluations of the synapse. a, Real-time PSC changes of the synapse during repeated write/erase operations over 3000 cycles. **b–d**, Detailed plot of PSC changes at 0 s (**b**), 3000 s (**c**), and 6000 s (**d**) after measurement.

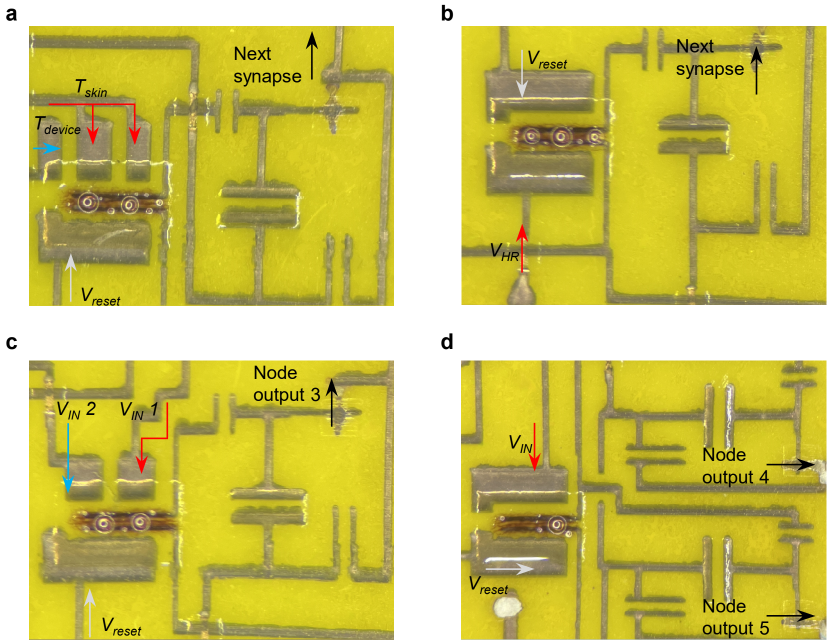

Supplementary Fig. 33 | Configurations of input and output terminals for each synapse-node integrated device. a–d, Input-output and reset configuration of synapse-node integrated processing unit for CBT calculation (**a**), heart-rate processing (**b**), synaptic AND logic (**c**), and biochemical sensing synapse (**d**).

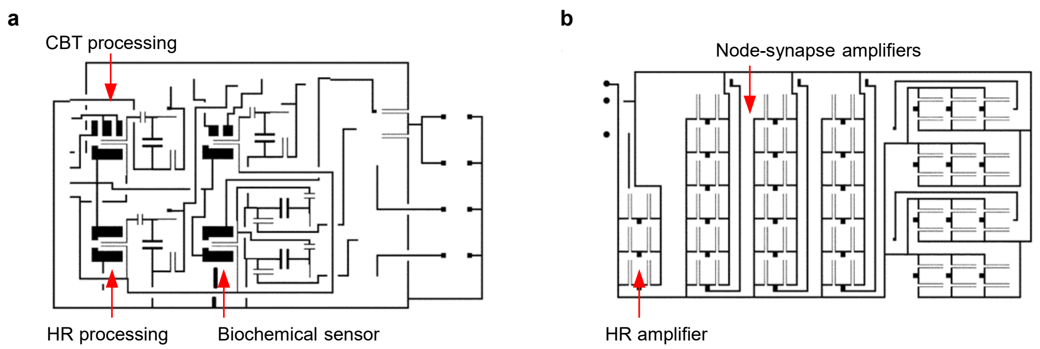

Supplementary Fig. 34 | Circuit diagram of wearable neuromorphic device for sepsis diagnosis. a, Design of the key parts of neuromorphic processing layer: CBT processing, HR processing, and biochemical sensor. **b**, The design of the complementary amplifier layer for HR signal amplification.

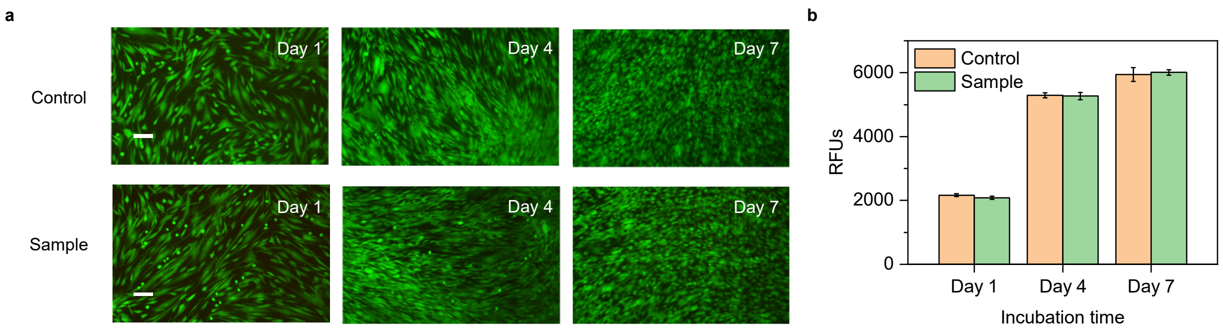

Supplementary Fig. 35 | Cytotoxicity of the wearable neuromorphic device. a, Representative live (green)/dead (red) images of human dermal fibroblasts (HDFs) cells cultured with the control (top) and test sample (bottom) after 1, 4, and 7 days. Scale bars, 100 μm. **b**, Quantitative measurement of cell metabolic activity during the 7-day culture period. RFUs indicate relative fluorescence units. Error bars represent the s.d. of the mean from 4 measurements.

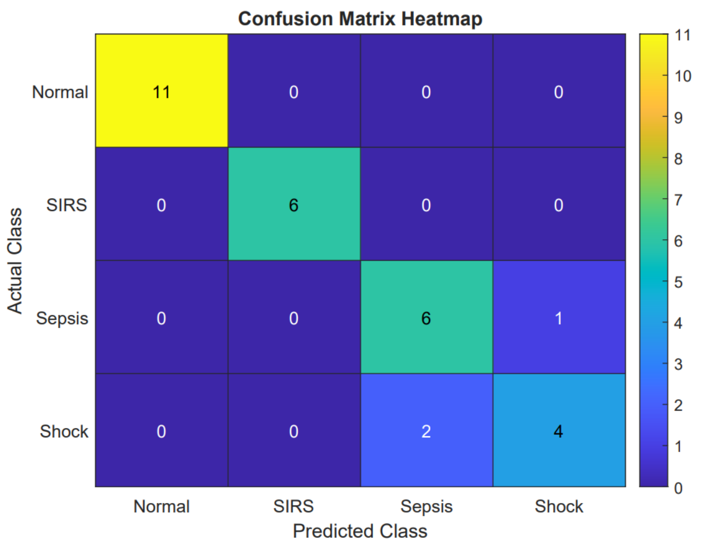

Supplementary Fig. 36 | Confusion matrix for a CSPINS validation. Total 32 cases including 6 SIRS, 7 SEPSIS and 6 SEPTIC SHOCK cases. Estimated accuracy was 84.4%.

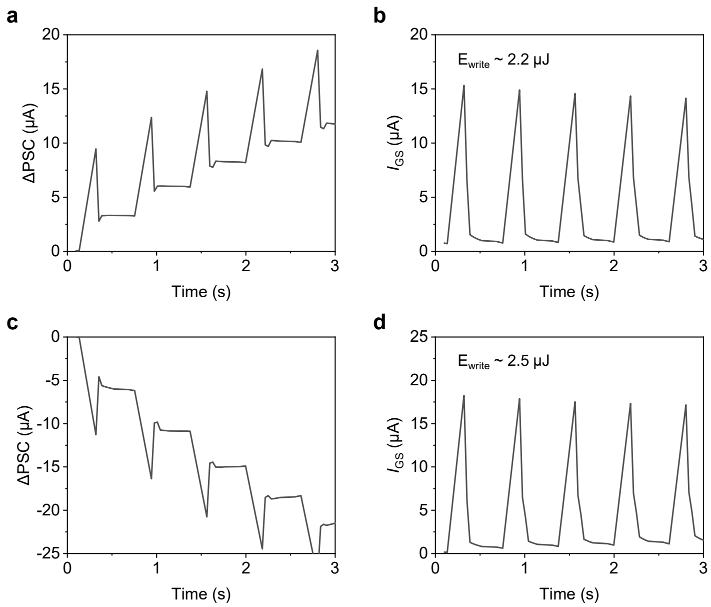

Supplementary Fig. 37 | Estimated energy consumption of the synapse. a,**b**, Real-time PSC changes of the synapse under repeated write operations (**a**) and the corresponding source-gate current plot and estimated power consumption during write operations (**b**). **c**,**d**, Real-time PSC changes of the synapse under repeated erase operations (**c**) and the corresponding source-gate current plot and estimated power consumption during the erase operations (**d**).

Supplementary Table 1 | List of material inks used in CSPINS fabrication.

Material	Solvent	Concentration (w%)	Viscosity (cP)	Nozzle Temp (°C)
Au	Water, Glycerol	9-11	3-5	37
Ag	Water, Glycerol	40	8-12	25
SWCNTs	CHP	5	10-14	30
MWCNTs	Water, EG	25	10-13	25
Nafion	EtOH, EG	8	6-8	25
MXene	Water, EG	1-2	3-5	25
cPVP	1-Hexanol	7	9-10	25
Ion-gel	PEGDA, IPA	88	16-18	35
SU-8	GBL	14	2-3	35
In_2O_3	2-ME	3	2-4	37

CHP, 1-cyclohexyl-2-pyrrolidone; EtOH, ethanol 200; EG, ethylene glycol; PGMEA, propylene glycol monomethyl ether acetate; PEDGA, poly(ethylene glycol) diacrylate; IPA, Isopropyl alcohol; GBL, gamma-butyrolactone; 2-ME, 2-methoxyethanol.

Supplementary Table 2 | Reference skin and device temperatures measured with commercial thermometer.

Location	Test number	T_{skin} (°C)	T_{AI} (°C)	$T_{reference}$ (°C)	R_{skin}/R_{device}
Forehead	1	33.8	31.5	36.5	1.2
	2	34.5	32.7	36.6	1.2
	3	34.6	32.8	36.6	1.1
	4	34.5	32.9	36.5	1.3
	5	34.5	32.9	36.5	1.3
Chest	1	34.1	31.8	36.8	1.2
	2	34.4	32.5	36.6	1.2
	3	34.5	32.6	36.6	1.1
	4	34.7	33.1	36.6	1.2
	5	34.8	33.3	36.6	1.2
Arm	1	32.2	29	35.9	1.2
	2	32.6	29.5	36.3	1.2
	3	33.2	30.5	36.1	1.1
	4	33.4	30.8	36.2	1.1
	5	33.5	31.3	36.4	1.3

T_{skin}, skin temperature; T_{AI}, device temperature; $T_{reference}$, armpit temperature measured by the commercial thermometer; R_{skin}/R_{device}, calculated ratio between heat resistivities of the skin and the device (PMDS spacer).

Supplementary Table 3 | List of selected diagnosis case for device validation. Biomarker data were extracted from selected patients diagnosed as systemic inflammatory response syndrome (SIRS), sepsis, and septic shock. For reference data, the values were set to representative values in healthy adult males.

Case No	Subject ID	Temp (°C)	HR (BPM)	Lactate (mM)	Diagnosis
1	Reference	36.5	80	1.0	Normal
2	164	38.3	94	1.5	SIRS
3	164	38.8	95	1	SIRS
4	164	37.6	116	1.2	SIRS
5	339	38.1	104	1.5	SIRS
6	339	38.3	107	0.8	SIRS
7	339	38.5	108	1.2	SIRS
8	305	38	113	3.9	Sepsis
9	605	37.7	100	2.2	Sepsis
10	605	37.9	106	2.1	Sepsis
11	689	39.3	91	2.1	Sepsis
12	711	37.9	119	2.1	Sepsis
13	975	37.7	122	3.4	Sepsis
14	1331	38.3	128	2	Sepsis
15	1331	37.9	125	2.7	Sepsis
16	305	37.8	110	6.3	Shock
17	605	38.1	108	4.8	Shock
18	711	37.7	95	4.4	Shock
19	975	37.9	108	12.4	Shock
20	1331	38.4	183	3.5	Shock
21	1331	38.3	191	2.7	Shock

Supplementary Table 4 | Synaptic current and node outputs under various case inputs.

Case No	Temp-Synapse	HR-Synapse	Lac-Synapse	SIRS	Sepsis	Shock	Normal
1	16.116	15.425	6.701	0	0	0	1
2	17.864	17.791	8.488	1	0	0	0
3	18.402	17.960	6.701	1	0	0	0
4	17.151	21.509	7.416	1	0	0	0
5	17.656	19.481	8.488	1	0	0	0
6	17.864	19.988	5.986	1	0	0	0
7	18.077	20.157	7.416	1	0	0	0
8	17.553	21.002	17.065	1	1	1	0
9	17.250	18.805	10.990	1	1	0	0
10	17.451	19.819	10.632	1	1	0	0
11	18.965	17.284	10.632	1	1	0	0
12	17.451	22.016	10.632	1	1	0	0
13	17.250	22.523	15.278	1	1	0	0
14	17.864	23.537	10.275	1	1	0	0
15	17.451	23.030	12.777	1	1	0	0
16	17.351	20.495	25.642	1	1	1	0
17	17.656	20.157	20.281	1	1	1	0
18	17.250	17.960	18.852	1	1	1	0
19	17.451	20.157	47.442	1	1	1	0
20	17.970	32.833	15.635	1	1	0	0
21	17.864	34.185	12.777	1	1	0	0

Supplementary Table 5 | List of selected confusion cases for device validation. Biomarker data were extracted from patients diagnosed with hypertension (high blood pressure, HBP), diabetes (DM) or both.

Case No	Subject ID	Temp (°C)	HR (BPM)	Lactate (mM)	Diagnosis
22	124	37.0	77	1.3	HBP
23	188	36.6	79	4.8	HBP, DM
24	199	37.5	78	1.4	DM
25	209	36.6	73	0.9	HBP, DM
26	222	36.0	79	2.3	HBP
27	236	36.4	100	1.8	HBP
28	518	38.3	82	1.4	DM
29	533	37.2	69	1.3	DM
30	890	35.4	102	3.5	DM
31	1767	36.8	78	2.3	DM
32	2090	37.5	82	2.9	HBP, DM

Supplementary Table 6 | Synaptic current and node outputs for various confusion case inputs. Simulation results of wearable neural network, detailing synaptic current response and corresponding node outputs.

Case No	Temp-Synapse	HR-Synapse	Lac-Synapse	SIRS	Sepsis	Shock	Normal
22	16.574	14.918	7.773	0	0	0	1
23	16.206	15.256	20.281	0	0	0	1
24	17.053	15.087	8.131	0	0	0	1
25	16.206	14.242	6.344	0	0	0	1
26	15.678	15.256	11.347	0	0	0	1
27	16.027	18.805	9.560	0	0	0	1
28	17.865	15.763	8.131	0	0	0	1
29	16.763	13.566	7.773	0	0	0	1
30	15.175	19.143	15.636	0	0	0	1
31	16.389	15.087	11.347	0	0	0	1
32	17.053	15.763	13.491	0	0	0	1

Supplementary Table 7 | Evaluation of energy consumption of entire CSPINS.

Component	Read current (μA)	Operation voltage (V)	Resistance (kΩ)	Pulse number	Energy (μJ)	Power (μW)
Chemical synapse	15.0	3.0	-	6.0	13.5	4.5
CBT (synapse)	15.0	3.4	-	6.0	15.3	5.1
HR (synapse)	15.0	3.5	-	6.0	15.8	5.3
CBT (sensor)	-	7.5	11.0	-	15340.9	5113.6
HR (sensor)	-	10.0	24.0	-	12500.0	4166.7
Synaptic node	0.02	0.15	-	1.00	0.0001	0.0008
Amplifier	-	10.0	3000.0	-	100.0	33.3

The power dissipation of the connected sensors including the CBT sensor for core body temperature (CBT) calculation and the HR sensor for heart rate data processing using the equation '$P_{total} = P_1 + P_2 = I_2 \times (R_1 + R_2) = V_{in}^2 / (R_1 + R_2)$'.

Supplementary Table 8 | Comparison of the power consumption of CSPINS with previously reported wearable sensor-processor integration systems targeting various biomarkers.

Reference	Target biomarkers	Power Consumption
This work	Lactate, Core-body temperature, Heartrate	~ 5.3 mW
1	Glucose, Lactate, Na^+, K^+	~ 92 mW
2	Glucose, Lactate, pH, Temperature	~ 55 mW
3	Alcohol , Glucose	~ 36 mW
4	Temperature, Heartrate, Motion	~ 274 mW
5	Heartrate	~ 400 mW

References

1. Gao, W. *et al.* Fully integrated wearable sensor arrays for multiplexed in situ perspiration analysis. *Nature* **529**, 509–514 (2016).
2. Choi, Y. S. *et al.* A transient, closed-loop network of wireless, body-integrated devices for autonomous electrotherapy. *Science* **376**, 1006–1012 (2022).
3. Kim, J. *et al.* Simultaneous Monitoring of Sweat and Interstitial Fluid Using a Single Wearable Biosensor Platform. *Adv. Sci.* **5**, 1800880 (2018).
4. Li, H., Sun, G., Li, Y. & Yang, R. Wearable Wireless Physiological Monitoring System Based on Multi-Sensor. *Electronics* **10**, (2021).
5. Majumder, A. J. A., ElSaadany, Y. A., Young Jr., R. & Ucci, D. R. An Energy Efficient Wearable Smart IoT System to Predict Cardiac Arrest. *Adv. Hum.-Comput. Interact.* **2019**, 1507465 (2019).
